# A comparative meta-analysis between chevron and scarf osteotomies in hallux valgus patients

**DOI:** 10.3389/fsurg.2025.1665319

**Published:** 2025-12-12

**Authors:** Turki Fahid Alqahtani

**Affiliations:** Anesthesia and Surgery Department, College of Medicine, Imam Mohammad Ibn Saud Islamic University (IMSIU), Riyadh, Saudi Arabia

**Keywords:** chevron, scarf, osteotomy, hallux valgus, patients

## Abstract

**Background:**

Usually affecting the medial prominence of the first metatarsophalangeal (MTP) joint, hallux valgus is a complicated malformation of the first ray that causes deformed joint structure, dysfunction, and increasing stiffness. The most common methods for treating hallux valgus malformation are scarf osteotomy and chevron osteotomy. Due to the inconsistent and contradictory findings among the studies, we conducted this systematic review and meta-analysis to compare chevron and scarf osteotomies in the management of hallux valgus deformity.

**Methods:**

Using the following search strategy: “Chevron” AND “Scarf” AND “Osteotomy” AND “Hallux Valgus”, and from inception until October 2024, we searched PubMed, Web of Science, and Scopus for relevant publications that needed to be screened to see if they could be included in our study. We performed a meta-analysis of the articles included using Review Manager version 5.4 software, pooling the mean difference (MD) of various outcomes at 95% confidence intervals (CI) and a *p*-value of 0.05.

**Results:**

Chevron osteotomy was observed to lower the hallux valgus angle (HVA) with a significant difference compared with scarf osteotomy, showing a MD = −2.44 (95% CI: −4.57, −0.31, *p* = 0.03). However, no significant difference was observed between both osteotomies regarding the reduction of intermetatarsal angle (IMA), showing a MD = −0.33 (95% CI: −1.32, 0.66, *p* = 0.52). Chevron osteotomy was observed to be associated with higher American Orthopedic Foot and Ankle Society (AOFAS) compared with scarf osteotomy with MD = 2.21 (95% CI: 0.7, 3.71, *p* = 0.004) and I^2^ = 0%, however, no significant difference was observed regarding their effect on pain with SMD = −0.07 (95% CI: −0.44, 0.31, *p* = 0.73).

**Conclusion:**

Chevron osteotomy was observed to be superior to scarf osteotomy in lowering the HVA and improving functional outcomes presented by AOFAS measurements. However, they were comparable in their effect on IMA and pain measurements.

## Introduction

Usually affecting the medial elevation of the first metatarsophalangeal (MTP) joint, hallux valgus is a complicated anatomic abnormality of the first ray that causes deformed joint engineering, malfunction, and increasing suffering. Hallux valgus is a prevalent disorder, affecting around 23% of persons aged 18–65 and 35.7% of individuals over 65. The frequency is greater in females, with literature reporting ratios that vary from 2:1–15:1, indicating the number of infected females per male (1–3).

The stability of the first ray depends on a number of both passive and active features at the first MTP and first tarsometatarsal (TMT) joints. It is thought that the first ray's medial structural elements weaken during the early stages of hallux valgus defect, causing the first metatarsal to medially deviate and the hallux to pronate and deviate laterally. This causes a progressive varus defect at the first TMT joint. The metatarsal head's position with the sesamoid apparatus changes as it spins in the frontal plane and shifts medially. The lateral sesamoid is located in the first intermetatarsal gap, and the first metatarsal head now rests on the medial sesamoid. Additionally, as the misplaced abductor hallucis plantar flexes and pronates the phalanx, the deformation at the MTP joint allows the hallux flexor and extensor tendons to bowstring laterally, adding to the deforming tension (4–6).

Scarf osteotomy and chevron osteotomy are the predominant techniques for correcting hallux valgus deformity. Certain writers assert that the scarf method is superior (7, 8), while others indicate improved outcomes with chevron osteotomy (9, 10), and some report equivalent results with both techniques (11–13). The chevron technique necessitates a reduced access area and a less extensive osteotomy, while the scarf technique demands a wider incision, a more prolonged osteotomy, increased soft-tissue resection, and more comprehensive internal fixation (9, 10, 14, 15). A reduced surgical duration distinguishes the chevron method, and it is regarded by some as a more straightforward surgical technique (9, 10). Certain publications indicate that chevron osteotomy may yield just a modest degree of hallux valgus correction, while scarf osteotomy facilitates a more comprehensive correction (11, 13).

Although several meta-analyses and systematic reviews have compared chevron and scarf osteotomies, most have been limited by older data, small numbers of randomized trials, heterogeneity in surgical techniques, and weak reporting of functional outcomes. For example, Peng et al. (16) restricted their analysis to RCTs published through 2018 and did not meaningfully stratify by deformity severity. Similarly, the 2019 Chinese meta-analysis by Deng et al. (17) focused on radiographic correction and lacked detailed functional analyses. A more recent meta-analysis comparing osteotomy sites (18) suggested no significant differences but was conducted in populations with predominantly mild to moderate deformities and did not specifically isolate chevron vs. scarf in diverse clinical settings. Given the publication of several newer comparative cohort studies and advances in surgical technique over the past 5–10 years, there is a clear need for an updated, more comprehensive synthesis. Our meta-analysis includes a broader range of evidence (randomized trials + observational studies) up to October 2024, stratifies results by deformity severity and surgical variant, and provides more detailed reporting on functional outcomes. This updated evidence base is essential to guide surgical decision-making in hallux valgus management. Therefore, we conducted this systematic review and meta-analysis to compare chevron and scarf osteotomies in the management of hallux valgus deformity.

## Methods

At every stage, following the Cochrane Handbook of Systematic Reviews of Interventions (19), we performed this systematic review and meta-analysis in accordance with the Preferred Reporting Items for Systematic Reviews and Meta-Analyses (PRISMA) statement's recommendations (20).

### Database searching

A comprehensive literature search was performed in PubMed, Scopus, and Web of Science from inception to October 2024, without language restrictions. The following key terms and Boolean combinations were used: *(“hallux valgus” OR “bunion”) AND (“scarf osteotomy” AND “chevron osteotomy” OR “distal metatarsal osteotomy”)*.

References of all eligible articles and relevant reviews were screened to identify additional studies.

### Screening

Following database searching, we used EndNote version 7 software to eliminate duplicates from the generated articles (21). Then, we transferred the rest of the content to the (22) software 19 to carry out the screening procedure (*Rayyan*). Two separate writers first screened the included papers by title and abstract to determine their eligibility for inclusion. Then they screened the full-text of the included articles from the first phase. Any disagreements were resolved by consensus or by consulting a senior author.

### Inclusion and exclusion criteria

Inclusion criteria followed the PICO framework: Population (P): Patients with symptomatic hallux valgus undergoing surgical correction, Intervention (I): Scarf osteotomy (with or without fixation), Comparator (C): Chevron osteotomy, and Outcomes (O): Radiographic [hallux valgus angle (HVA), and intermetatarsal angle (IMA)] and clinical (AOFAS, and pain). We included randomized controlled trials (RCTs) and observational cohort studies that compared chevron and scarf osteotomies in patients with hallux valgus. Exclusion criteria included: (1) non-comparative studies, (2) cadaveric or biomechanical-only analyses, (3) reviews or conference abstracts, and (4) studies lacking quantitative data for main outcomes.

Primary outcomes were: (1) change in HVA, (2) change in IMA, and (3) postoperative AOFAS scores. Secondary outcomes included pain scores.

### Quality assessment

We evaluated the quality of the included observational cohort studies using the Cochrane Newcastle Ottawa scale (NOS) instrument. With the exception of the comparison question, which can receive two stars, each of the eight questions has a maximum score of one star. As a result, nine is the greatest possible score and zero is the lowest. Studies with a score of 0–3 were deemed low quality, those with a score of 4–6 were deemed moderately good, and those with a score of 7–9 were considered highly good (23). For RCTs, we evaluated bias in the included trials using the Cochrane Risk of Bias 2 tool (Rob-2) (24).

### Data extraction

The baseline data were extracted, including study design, sample size, age, and gender, as well as the outcomes, such as baseline and posttreatment values of intermetatarsal angle (IMA), hallux valgus angle (HVA), and the American Orthopedic Foot and Ankle Society (AOFAS), as well as pain metrics following surgery. This was done using Microsoft Excel sheets by two independent authors.

### Statistical analysis

By pooling the mean difference (MD) of the change from baseline comparing the two surgical methods in IMA, HVA, and AOFAS and posttreatment pain at 95% confidence intervals (CI) and a *p*-value of 0.05, we performed a meta-analysis of the articles included using Review Manager version 5.4 software. Heterogeneity was tested using the I^2^ and the *p*-value for significance.

Because of anticipated heterogeneity in patient characteristics and follow-up durations, a random-effects model (DerSimonian–Laird method) was used for all pooled analyses. Standardized mean difference (SMD) was used for different scales. Statistical heterogeneity was quantified using the I² statistic, with thresholds of 25%, 50%, and 75% indicating low, moderate, and high heterogeneity, respectively.

## Results

### Database searching and screening

**Figure 1:**
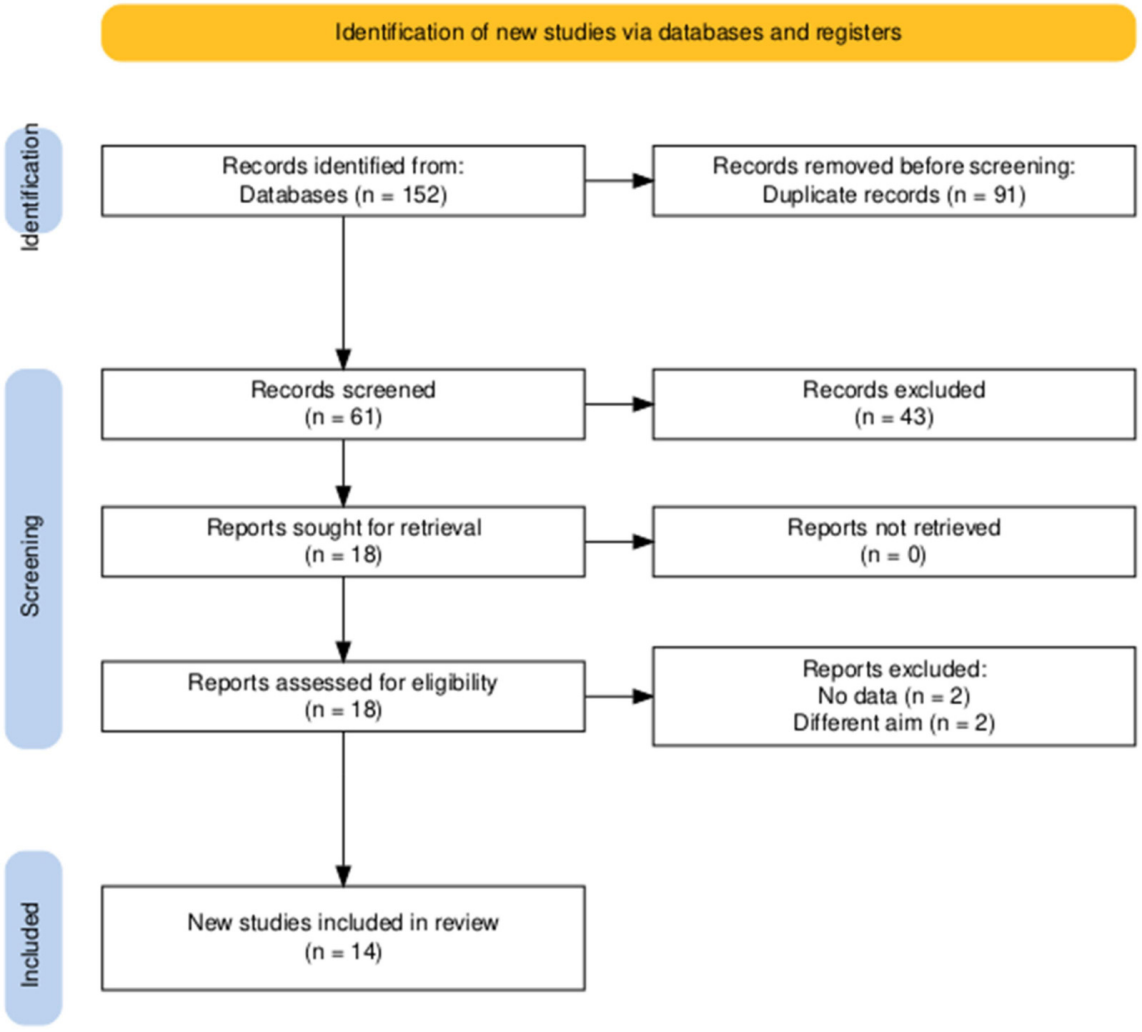
PRISMA flow diagram of searching and screening processes.

A total of 61 papers made it through the title and abstract screening after the database search produced 152 articles altogether, including 91 duplicates. Fourteen articles were obtained after 43 were eliminated and eighteen were vetted by full-text (7, 9–12, 14, 25–32) in the current systematic review and meta-analysis (Figure 1).

### Quality and risk of bias assessment

**Figure 2:**
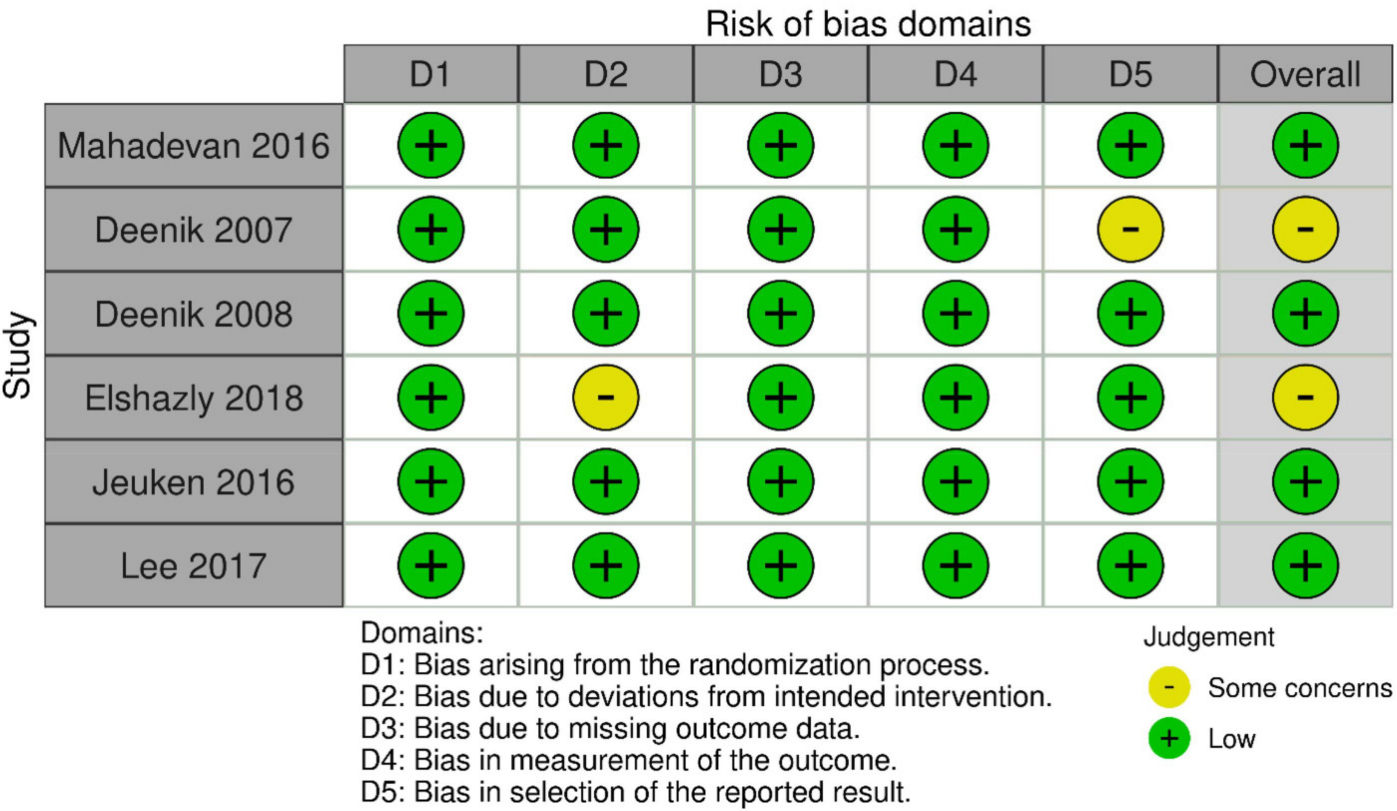
Risk of bias assessment of RCTs using Rob-2.

Four of the included RCTs were deemed to have low risk of bias, and the other two had some concerns according to Rob-2 (Figure 2). While six of the cohort studies had high quality, and two of them had moderate quality according to NOS (Table 1).

**Table 1:** Quality assessment of cohort studies using NOS.

Study name	The level of representation of the exposed population (★)	Choice of the unexposed cohort (★)	Determination of exposure (★)	Evidence that the outcome of interest was absent at the commencement of the research (★)	Similarity of cohorts based on design or analysis (max★★)	Was the duration of follow-up sufficient for the consequences to manifest? (★)	Evaluation of results (★)	Assessment of cohort follow-up adequacy (★)	Quality level
Kulinski et al. (28)	☆	☆	☆	☆	☆	☆	☆	☆	High
Wu et al. (32)	☆	-	☆	☆	☆	☆	☆	☆	High
Fakoor et al. (7)	☆	-	☆	☆	☆	☆	☆	☆	High
Choi et al. (25)	☆	-	-	☆	☆	☆	☆	☆	Moderate
Frigg et al. (27)	☆	☆	☆	☆	☆	☆	☆	☆	High
Lai et al. (9)	☆	☆	☆	☆	☆	☆	☆	☆	High
Tay et al. (30)	☆	☆	☆	☆	☆	☆	☆	☆	High
Vopat et al. (31)	☆	-	☆	☆	☆	☆	☆	-	Moderate

### Baseline characteristics

We included 14 studies comparing 677 patients undergoing chevron osteotomy against 570 patients undergoing scarf osteotomy for hallux valgus treatment. Six of the included studies were RCTs, and eight of them were cohort studies. The mean age of participants ranged from 36–62.35 years old (Table 2).

**Table 2:** Baseline characteristics of the included studies.

Study ID	Study design	Sample size		Age, mean (SD)		Male, *n* %)	
		Chevron	Scarf	Chevron	Scarf	Chevron	Scarf
Mahadevan et al. (10)	RCT	60	49	50.7 (14.1)		9 (10.7)	
Deenik et al. (11)	RCT	47	49	43	45	NR	NR
Deenik et al. (26)	RCT	70	66	NR	NR	NR	NR
Elshazly et al. (12)	RCT	22	21	36 (12.16)		19 (44)	
Jeuken et al. (14)	RCT	37	36	56.3 (13.9)	58.2 (14.1)	4 (10.8)	2 (5.5)
Kulinski et al. (28)	Cohort	181	32	62.35	60.66	15 (7)	
Wu et al. (32)	Cohort	60	40	47.51	46.5	NR	NR
Fakoor et al. (7)	Cohort	23	10	35.6	41.7	3 (13)	1 (10)
Choi et al. (25)	Cohort	27	52	44.3 (14.6)	47.5 (13.2)	NR	NR
Frigg et al. (27)	Cohort	48	50	48.04 (13.2)	48.23 (12.1)	7 (14.6)	6 (12)
Lai et al. (9)	Cohort	29	58	54.3 (12.8)	54.3 (12.7)	4 (13.8)	6 (10.3)
Lee et al. (29)	RCT	25	25	52.6 (14)	53.4 (12.5)	2 (8)	3 (12)
Tay et al. (30)	Cohort	30	30	51.7 (13.6)	52.7 (14.3)	9 (30	4 (13.3)
Vopat et al. (31)	Cohort	18	52	58.7	56.5	1 (5.5)	7 (13.5)

RCT, randomized controlled trial; NR, not reported; SD, standard deviation.

**Figure 3:**
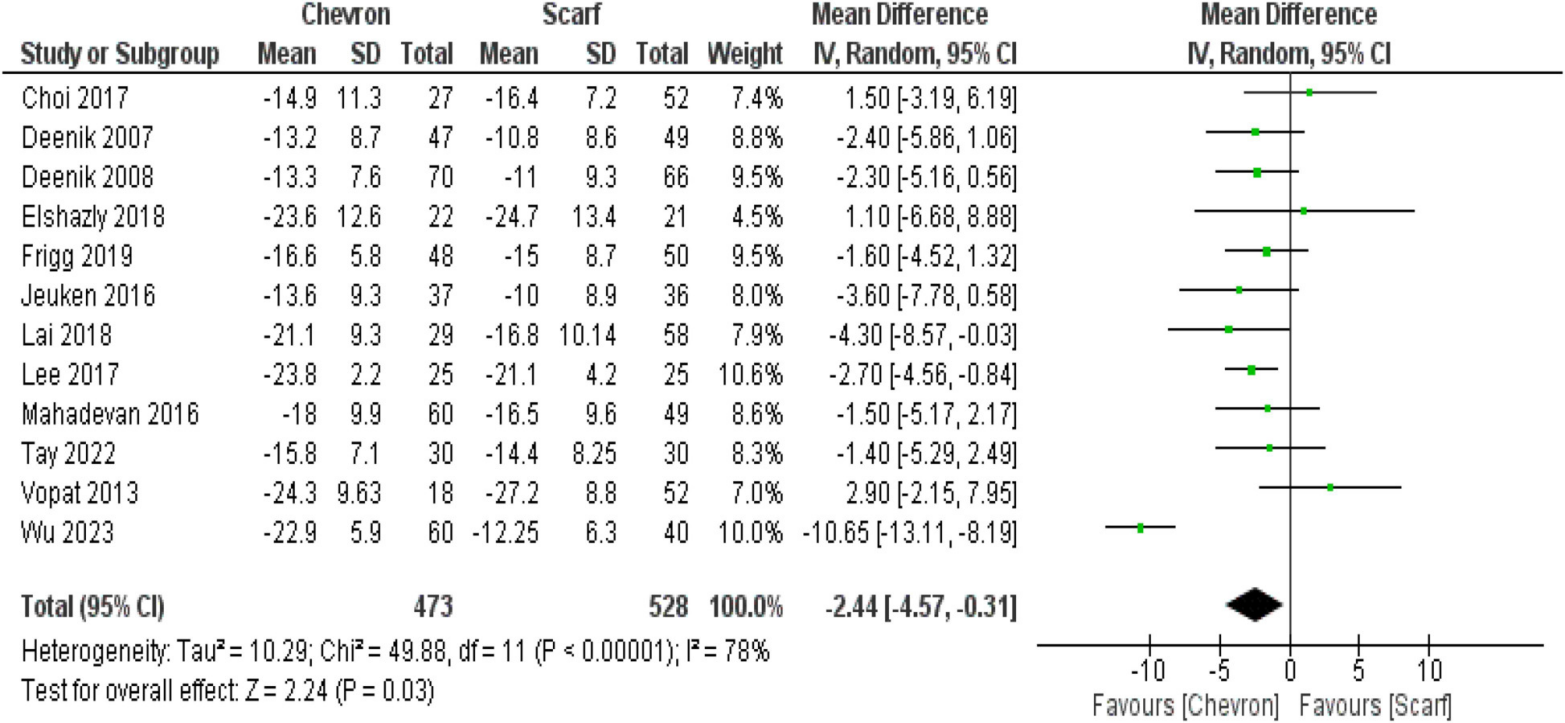
Comparison between Chevron and Scarf osteotomies in the reduction of HVA in hallux valgus patients.

Chevron osteotomy was observed to lower HVA with a significant difference compared with scarf osteotomy, showing an MD −2.44 (95% CI: −4.57, −0.31, *p* = 0.03), and I^2^ 78%, *p* < 0.00001. However, no significant difference was observed between both osteotomies regarding the reduction of IMA, showing a MD = −0.33 (95% CI: −1.32, 0.66, *p* = 0.52) (Figure 3 and Supplementary Figure S1).

Chevron osteotomy was observed to be associated with higher AOFAS compared with scarf osteotomy with MD = 2.21 (95% CI: 0.7, 3.71, *p* = 0.004) and I^2^ = 0%, however, no significant difference was observed regarding their effect on pain with SMD = −0.07 (95% CI: −0.44, 0.31, *p* = 0.73) (Supplementary Figures S2, S3).

## Discussion

The present comprehensive review and meta-analysis evaluated chevron and scarf osteotomies in patients with hallux valgus. The results demonstrated the superiority of chevron osteotomy in reducing HVA compared to scarf osteotomy, along with enhancements in AOFAS metrics, while exhibiting similar effects on IMA and pain levels.

Our pooled analysis (14 studies, 1,247 feet) found that chevron osteotomy produced a modestly greater reduction in HVA than scarf osteotomy (MD −2.44; 95% CI −4.57 to −0.31), while IMA and pain were similar between procedures; AOFAS favored chevron by a small margin (MD 2.21). These results are broadly consistent with prior RCT-only meta-analyses (16, 17), which also reported superior HVA correction with chevron but no clear differences in IMA, function, or complications. Other pooled work that compared osteotomy sites (18) reported no consistent advantage between distal (chevron) and mid-shaft (scarf) osteotomies in mostly mild-to-moderate deformities, underscoring the impact of study selection, deformity severity, and surgical variants on pooled estimates. Taken together, the literature indicates a reproducible radiographic advantage for chevron in HVA correction, but uncertainty remains regarding clinically important functional benefits and complication profiles. Also, the number of included studies in previous meta-analyses was small, and some of them included RCTs only. Therefore, we conducted this current meta-analysis, including RCTs and cohort studies, and investigated different outcomes.

Orthopedic doctors lack unanimity about the superiority of the scarf over chevron osteotomy for the repair of hallux valgus deformity (8, 11, 12, 14, 15, 31, 33). The objective of hallux valgus correction is to enhance aesthetics and functionality, alleviate pain, and avoid recurrence (9, 12, 31, 34).

Mahadevan et al. (10) implemented a straightforward alteration of the chevron osteotomy angle to get an elongated plantar limb. By increasing the area of contact, this enhanced stability and allowed for more movement. To reduce the risk of harming the plantar nutrition veins supplying the metatarsal head, the plantar incision was positioned away from the distal widening of the metatarsal bone (35), this so lowers the chance of osteonecrosis. There was no evidence of avascular necrosis in this case (10).

The term “scarf” originates from a construction method in which two wooden pieces are interconnected with their longer closes overlapped, thereby enhancing stability against tension and compression power (36). Traditionally, the scarf osteotomy was executed through either transformation or rotation of the osteotomy segment, with the latter method employed for malformations exhibiting a higher IMA (37). Lopez et al. (38) delineate the translating and rotating procedure, referred to as “trotation” scarf osteotomy. This osteotomy is frequently performed in conjunction with an Akin osteotomy, a phalangeal, a phalangeal adductory osteotomy (Akin procedure), to improve the lateral deviation component of the hallux valgus deformity (39). In the traditional scarf technique, the dorsal and plantar osteotomy cuts are kept approximately parallel to maintain stable translation; however, modifications such as the Maestro technique permit controlled divergence of the cuts, which can achieve effective translation and correction without compromising stability (40). The modified chevron osteotomy, characterized by its simpler extended plantar limb and shorter design with a single dorsal step-cut, is thought to circumvent these issues and may elucidate the enhanced IMA correction noted by Mahadevan et al. (10).

A Scarf osteotomy has numerous downsides and limits. Barouk (41) indicated a significant learning curve, with total problems in previous studies varying from 6%–35% (42, 43). Intraoperative troughing is the primary cause of the elevated complication rate, which can be mitigated; thus, we regard the entire complication rate as being on the lower end of this spectrum in recent years. Nonetheless, troughing is a significant problem leading to a rotational malunion of the first metatarsal, which is challenging to rectify. Smith et al. (43) documented first metatarsal fractures in 3% of cases linked to inadequate osteotomies of the first metatarsal or excessive force during screw insertion.

Complications and insufficient correction exacerbate surgical treatment results. The incidence of complications significantly influences the choice of treatment strategy, as managing foot issues frequently poses challenges for orthopedic surgeons and necessitates further surgical interventions (34, 44–46). Complications have been reported subsequent to both chevron and scarf osteotomy (44, 46–48). Chevron osteotomies exhibit elevated incidences of the most severe complication, avascular (aseptic) necrosis of the first metatarsal head, when contrasted with scarf osteotomies (11, 31). Furthermore, scarf osteotomies facilitate enhanced fixation of bone fragments, resulting in reduced rates of fixation instability and nonunion (13, 48, 49). Certain surgeons employ modified chevron osteotomy to rectify severe hallux valgus abnormalities (31). Furthermore, certain writers assert that the scarf technique facilitates the treatment of more significant abnormalities (10, 11). However, this process presents increased technical obstacles owing to its elevated complexity and requisite precision (50).

A further significant element in choosing the treatment for hallux valgus deformity correction is its cost. Chevron osteotomy is an additional cost-effective treatment alternative to scarf osteotomy (10, 51). Moreover, the screwless scarf modification further enhances cost-effectiveness by obviating the need for internal fixation while maintaining stable correction (40, 52). Furthermore, worse adjacent-joint arthritis subsequent to a hallux valgus correction technique may result in increased pain intensity, restricted foot mobility, and deteriorated limb functionality (53, 54).

Miller et al. (55) documented overcorrection in 1.4% of patients who had scarf osteotomy, while Choi reported this issue in 3.8% of patients (56). Various authors documented a loss of correction in 2%–73% of chevron cases and in 2%–75% of scarf cases (12, 14, 34). A significant report regarding loss of correction is presented in a study by Jeuken et al. (14), which noted a more pronounced loss of correction compared to other researchers. The authors recorded a loss of adjustment in 75% of scarf osteotomies and 73% of chevron osteotomies (14). Miller et al. (55) documented a single instance (1.4%) of fixation instability within the scarf osteotomy cohort. Choi reported the necessity of fixation removal in 4 cases (7.54%) (56), whereas Malatray et al. (47) reported it in 5 cases (10%). Potenza et al. (48) described metatarsal head necrosis after chevron osteotomy in 1 case (1.9%), while Deenik et al. (11) documented it in 3 cases (6.4%). Fixation removal was required in 3 instances (2.9%) among the chevron osteotomy patients assessed by Liszka et al. (44). Consequently, the problems arising from both surgeries differ across research, potentially influenced by the patients' conditions and the surgeon's expertise.

Beyond numerical comparisons, biomechanical and surgical factors influence the clinical choice between scarf and chevron osteotomies. The scarf osteotomy provides a long biplanar cut, allowing greater correction power and rotational adjustment with inherent mechanical stability. It can address moderate-to-severe deformities but has a steeper learning curve and may be technically demanding, particularly regarding accurate cut orientation and fixation placement. In contrast, the chevron osteotomy is technically simpler, with shorter operative time and a lower risk of troughing, making it favorable for mild-to-moderate deformities. However, its correction power is limited, and stability depends on precise alignment or screw fixation. These biomechanical distinctions explain variations in postoperative correction and recurrence rates reported in the literature. Complication patterns also differ: scarf osteotomy more frequently involves fixation removal or overcorrection, while chevron osteotomy is associated with mild residual deformity or limited correction in severe cases (16, 57).

The current study is a comprehensive meta-analysis comparing the scarf and chevron osteotomies in hallux valgus and investigating various functional and clinical parameters. However, there exist some limitations, including the difference in study design, as the observational studies may introduce bias for the results, different techniques in the studies, and a small sample size in most of the studies. Comparison between the two techniques in the risk of complications was not presented in comparative studies adequately for the analysis (7, 9, 12, 28), therefore this should be more focused on in the future studies.

## Conclusion

Chevron osteotomy was observed to be superior to scarf osteotomy in lowering the HVA and improving functional outcomes presented by AOFAS measurements. However, they were comparable in their effect on IMA and pain measurements.

## Data Availability

The original contributions presented in the study are included in the article/Supplementary Material, further inquiries can be directed to the corresponding author.
