# Supplementary material for: A comparative meta-analysis between chevron and scarf osteotomies in hallux valgus patients
Source: Front Surg. 2025 Dec 12;12:1665319. doi: 10.3389/fsurg.2025.1665319 (PMC12741154; doi:10.3389/fsurg.2025.1665319)
Supplement: Supplementary file 1 [file Datasheet1.docx]

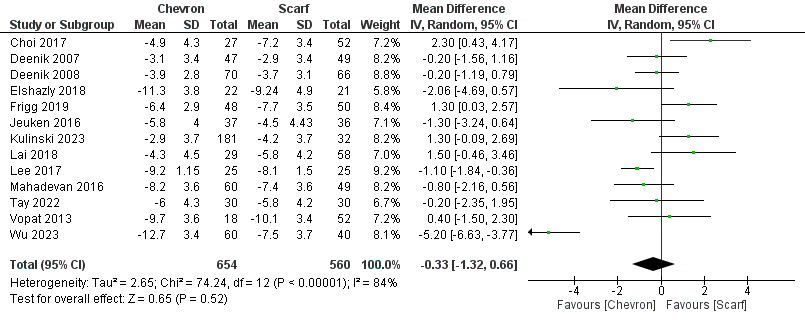


Figure S 1: Comparison between Chevron and Scarf osteotomies in reduction of IMA in Hallux Valgus patients


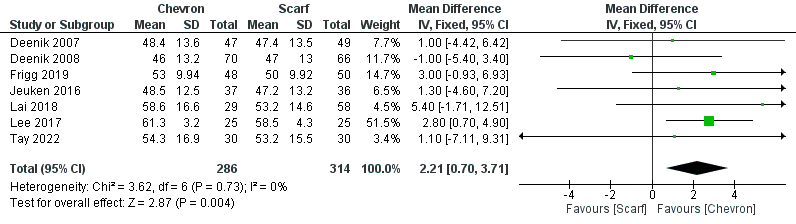


Figure S 2: Comparison between Chevron and Scarf osteotomies in the effect on AOFAS in Hallux Valgus patients


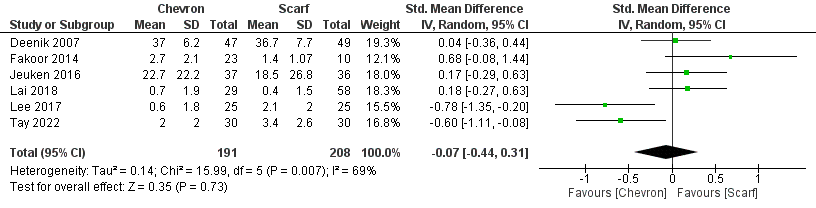


Figure S 3: Comparison between Chevron and Scarf osteotomies in the effect on pain in Hallux Valgus patients
